# Mesenteric Lipoblastoma and Cervical Lipoblastomatosis: Ultrasound, Elastosonography, and Computed Tomography Findings in Two Children

**DOI:** 10.1155/2014/478252

**Published:** 2014-03-27

**Authors:** Raffaella Capasso, Eugenio Rossi, Luisa Castelli, Antonio Basilicata, Raffaele Zeccolini, Massimo Zeccolini, Antonio Rotondo

**Affiliations:** ^1^Institute of Radiology, Second University of Naples (SUN), Piazza Miraglia 2, 80138 Naples, Italy; ^2^Department of Radiology, Santobono-Pausilipon-Annunziata Children's Hospital, 8012 Naples, Italy; ^3^Faculty of Medicine and Surgery, Second University of Naples, Primo Policlinico, Piazza Miraglia 2, 80138 Napoli, Italy

## Abstract

Lipoblastomas are benign tumors of the embryonic lipoid cells mainly occurring in infancy and early childhood. They are clinicopathologically distinguished in two forms: the well-circumscribed and localized type and the diffuse, irregularly confined type with infiltrative growth pattern, also called lipoblastomatosis. We report two pediatric cases of a mesentery localized and cervical diffuse lipoblastomas investigated both with ultrasound and computed tomography examinations.

## 1. Introduction

Lipoblastoma (LB) is a rare tumor of soft tissues which mainly occurs in infancy and early childhood [1–4]. It is usually located in the soft tissues of the limbs and trunk commonly presenting as a painless mass that can variably grow. Clinicopathologically LBs are distinguished into two benign forms: circumscribed or diffuse (lipoblastomatosis) [1, 2]. Despite its benign biological behavior LB may become symptomatic because of its enlargement and compression to adjacent organs and structures. The best treatment to prevent recurrences is the complete surgical excision [3, 4].

## 2. Case Report

### 2.1. Case 1

A 3-year-old male was brought to our institution suffering from abdominal pain and vomiting. An intra-abdominal painless palpable mass was appreciable and blood tests were normal.

**Figure 1:**
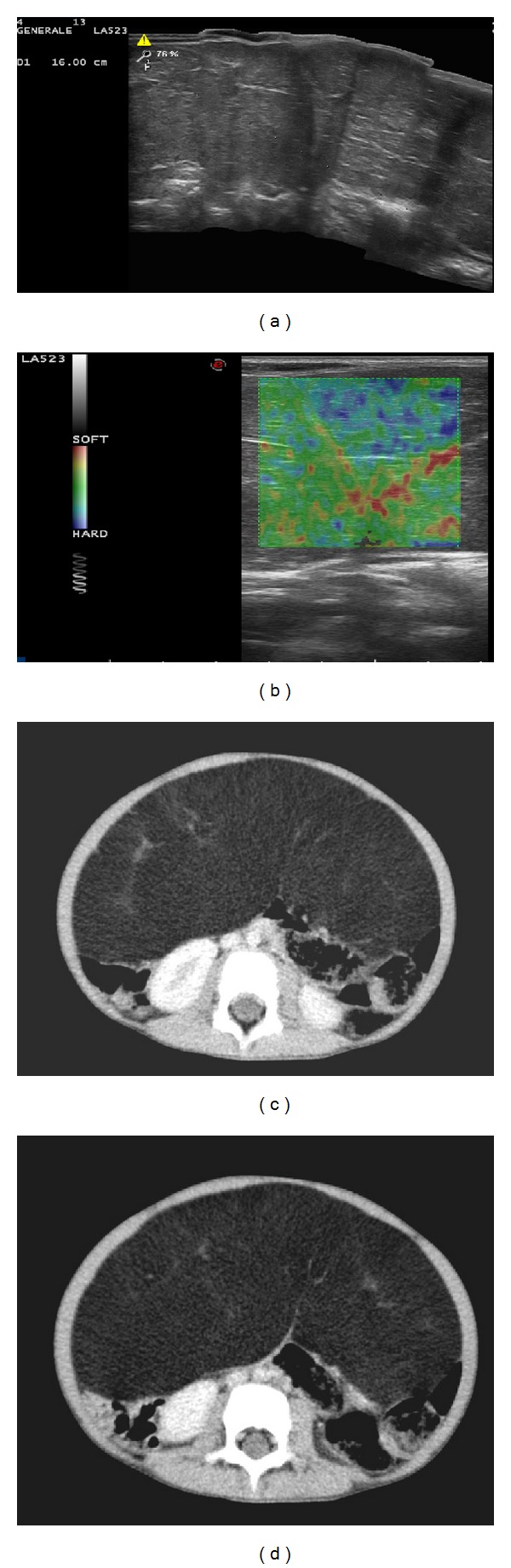
(a) US examination revealed mass had mixed echogenicity with some hyperechoic thin septations. (b) The mass appeared mainly green—soft—on elastosonography. ((c), (d)) CT showed the mass had fatty density with a few septations and no contrast enhancement.

Abdomen ultrasonography (US) showed a huge coarse mass of mixed echogenicity in paravertebral region dislocating pancreas and intestinal loops (Figure 1(a)). US color-Doppler evaluation stated a poor intra- and perilesional vascularity while elastosonography revealed mass had a soft consistency appearing green on colorimetric hardness scale (Figure 1(b)). Computed tomography (CT) scan displayed that the mass had adipose densitometry, was capsulated with some septa of soft-tissue attenuation, and extended from subhepatic space to the lower pelvis compressing intestinal loops. After intravenous contrast medium administration, the contrast enhancement was low and mainly appreciable in the septations (Figures 1(c) and 1(d)). The findings suggested a peritoneal lipomatous neoplasm. Child underwent surgery and through histological examination of surgical specimen the diagnosis of mesenteric LB was finally made.

### 2.2. Case 2

A 3-year-old girl underwent neck US at our institution because of a right cervical swelling departing from homolateral supraclavicular fossa. When the child was 6 months old, she was surgically treated for a cervical lipoblastomatosis which was not excised as a whole because of its expansion in depth. Thus she continued to complain from severe dyspnea.

**Figure 2:**
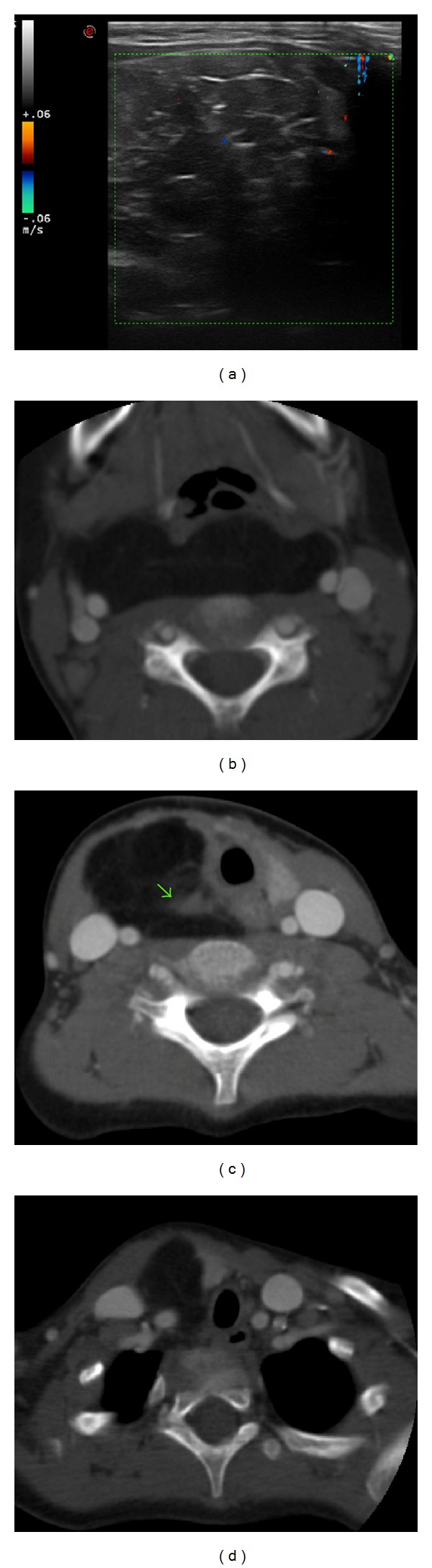
(a) US color-Doppler evaluation revealed low vascularity of the tumor. CT scan showed the extent of the fatty mass in (b) retropharyngeal space displacing the right vascular bound, (c) along the right side of the trachea, where along the right side of the trachea where an enhanced solid nodule (arrow) was appreciable an enhanced solid nodule (arrow), and (d) up to right supraclavicular fossa.

US examination revealed a slightly hyperechoic mass longitudinally extending with close adherence to the right wall of the trachea. The mass pushed forward the right lobe of thyroid gland and diverted laterally the great vessels of the neck. No vascularity of the mass was shown on color-Doppler evaluation (Figure 2(a)).

To better determine the extension of the mass, CT examination was further performed.

On CT scan the cervical mass appeared dishomogeneously hypodense with thin internal septations, extending from supraclavicular right fossa up to the retropharyngeal space, lining the trachea and reaching the great vessels of the upper left side of the neck. The mass exercised a compressive effect on adjacent structures, displacing the right cervical vasculonervous bundle, the trachea, and thyroid gland. Contrast enhancement was poor and limited to a solid nodule infiltrating the right side of trachea (Figures 2(b), 2(c) and 2(d)).

## 3. Discussion

LB is a rare, benign tumor of the embryonic lipoid cells presenting mostly in children and infants below 3 years of age, with no clear sex predominance [1–5]. It represents up to 30% of all adipose tumors which approximately amount to 6% of all soft tissue neoplasms in the pediatric population [5]. Clinicopathologically, LBs are classified in two forms: the well-circumscribed, capsular and localized type and the diffuse, irregularly confined and noncapsulating type with infiltrative growth pattern, also called lipoblastomatosis [1–3]. It has recently been suggested that this distinction may not be clinically relevant, because both circumscribed and infiltrative lipoblastomas can recur [3].

Less than 200 cases of LBs (localized and diffuse types) at various locations have been reported in literature: LB is mainly located in the soft tissues of the trunk and extremities, while it is rarely reported in the face, neck, buttock, perirectal area, and abdomen [1, 6].

Among rare localizations, occurrence at the mesentery is described in less than 20 cases until today showing a male predilection [1, 4]. The origin from adipose tissue of the neck represents about 10–15% of all reported LBs [6]. Most of them are located on the left or right side of the neck, while in our little patient LB extended to both sides crossing the cervical midline [6].

Although most LBs are asymptomatic at presentation, they can manifest, as in our cases, with a painless palpable mass and progressive symptoms of various organ compression according to the site of origin [3, 7]. As in Case 1, mesenteric origin can cause abdominal distention and abdominal pain; however it can manifest with bilious vomiting, loss of appetite, and diarrhea [4]. The rare localization in retropharyngeal space and the huge extension of the mass in Case 2 justify respiratory symptoms of the little girl.

For both our patients, CT densitometry and morphologic features suggested diagnosis. Indeed, the consistent radiologic feature of LB is the presence of fat within the lesion. It has been reported that in an infant or child, the most likely diagnosis for a well-circumscribed soft-tissue lesion that mainly contains fat is a LB [7].

The fatty nature of the two tumors was suspected at US exams because both masses showed features in agreement with LB typical US appearance: a homogeneously to finely textured, echogenic mass [7].

US elastography also confirmed the nonsolid nature of the mesenteric mass because the tumor appeared almost entirely green (soft) on hardness colorimetric scale. However US does not allow the total mass volume assessment often leading to underestimation of tumor size as clearly demonstrated by Case 2 [7].

Then, CT or magnetic resonance (MR) imaging is typically required to accurately demonstrate the complete extent of disease and for characterization also thanks to both CT and MR imaging ability to confirm the presence of fat within the lesion. LB usually appears as an encapsulated mass with fatty content and internal septa, showing absent to mild enhancement when it is mostly fat and marked and heterogeneous enhancement when fat component is limited [7].

Furthermore, MR imaging allows the evaluation of fat cells maturity through T1-weighted sequences because lipocytes have comparatively high signal intensity, whereas lipoblasts, typical for LB, have lower signal intensity [7]. Thus, LB can be heterogeneous on T1-weighted images having intermediate to high signal intensity according to the amount of immature lipoblasts [8]. Although MR imaging is described as the preferred tool for both diagnosis and preoperative evaluation of suspected LB, it is a more difficult and complex procedure to perform in children. Indeed, even if CT is burdened by the use of ionizing radiations, it is faster and more feasible than MRI when one should avoid sedation, as in our cases according to the will of children's parents. Our reported cases are an example of US and CT complementary roles in diagnosis and surgical planning of LB [6, 7].

In pediatric patients, the differential diagnosis for a fat-containing mass includes LB (circumscribed or infiltrative), teratoma, fatty overgrowth related to vascular malformations, lipoma, and liposarcoma. Lipoblastomatosis may show signs of muscular infiltration. Both lipomas and liposarcomas are very rare in young patients. Teratomas tend to contain calcification, which is not a feature of lipoblastoma [7]. However, imaging cannot differentiate between the different lipomatous tumors, which are radiologically indistinguishable. So, the only definitive procedure for diagnosing a soft-tissue mass is the histological analysis [1, 3, 4].

Some reports support that LBs undergo cellular maturation and may evolve into mature lipomas or spontaneously regress [7]. When LBs show rapid growth rate and huge size then wait and see; approach is usually precluded and the complete tumor removal is the treatment of choice [7]. After complete resection recurrence occurs in less than 25% of patients and metastases are not reported [7, 8]. For the little boy of Case 1 the complete excision of the tumor was obtained without any recurrence to date.

Although complete surgical excision is recommended to minimize local recurrence rates it has not been performed at the expense of disfiguring or debilitating surgery [4, 8]. If the entire tumor cannot be safely removed at the time of initial resection, a staged approach is recommended [3]. This staged approach has been employed for our little girl because the first surgery could not be radical and the child is still monitored in order to plan the next surgical reduction of the cervical mass [1].

Postoperative surveillance is important to detect relapses but there is no agreement on the appropriate length of followup for LB [5]. According to histologic benignity of LBs, with no metastatic potential, irradiation and chemotherapy are generally not employed [7].
